# Pregnane-Type Steroids from the Formosan Soft Coral *Scleronephthya flexilis*

**DOI:** 10.3390/ijms150610136

**Published:** 2014-06-06

**Authors:** Chao-Ying Kuo, Yung-Shun Juan, Mei-Chin Lu, Michael Yen-Nan Chiang, Chang-Feng Dai, Yang-Chang Wu, Ping-Jyun Sung

**Affiliations:** 1 Graduate Institute of Marine Biotechnology, National Dong Hwa University, Pingtung 944,Taiwan; E-Mails: recall04729@hotmail.com (C.-Y.K.); jinx6609@nmmba.gov.tw (M.-C.L.); 2 National Museum of Marine Biology and Aquarium, Pingtung 944, Taiwan; 3 Department of Urology, Kaohsiung Municipal Hsiao-Kang Hospital, Kaohsiung 812, Taiwan; E-Mail: juanuro@gmail.com; 4 Department of Urology, College of Medicine, Kaohsiung Medical University, Kaohsiung 807, Taiwan; 5 Department of Urology, Kaohsiung Medical University Hospital, Kaohsiung 807, Taiwan; 6 Department of Chemistry, National Sun Yat-sen University, Kaohsiung 804, Taiwan; E-Mail: michael@mail.nsysu.edu.tw; 7 Institute of Oceanography, National Taiwan University, Taipei 106, Taiwan; E-Mail: corallab@ntu.edu.tw; 8 School of Pharmacy, College of Pharmacy, China Medical University, Taichung 404, Taiwan; 9 Chinese Medicine Research and Development Center, China Medical University Hospital, Taichung 404, Taiwan; 10 Center for Molecular Medicine, China Medical University Hospital, Taichung 404, Taiwan; 11 Graduate Institute of Natural Products, Kaohsiung Medical University, Kaohsiung 807, Taiwan; 12 Department of Marine Biotechnology and Resources and Asia-Pacific Ocean Research Center, National Sun Yat-sen University, Kaohsiung 804, Taiwan

**Keywords:** soft coral, pregnane, *Scleronephthya flexilis*, cytotoxicity, MOLT-4

## Abstract

Three pregnane-type steroids, including a new metabolite, 3β-methoxy-5,20-pregnadiene (**1**) along with two known analogues, 3β-acetoxy-5,20-pregnadiene (**2**) and 5α-pregna-1,20-dien-3-one (**3**) were isolated from the soft coral *Scleronephthya flexilis*. Standard spectroscopic techniques were used to determine the structure of new steroid **1**. The absolute stereochemistry of steroid **2** was confirmed by X-ray diffraction analysis. Steroid **3** exhibited potent activity against MOLT-4 tumor cells.

## 1. Introduction

**Scheme 1:**
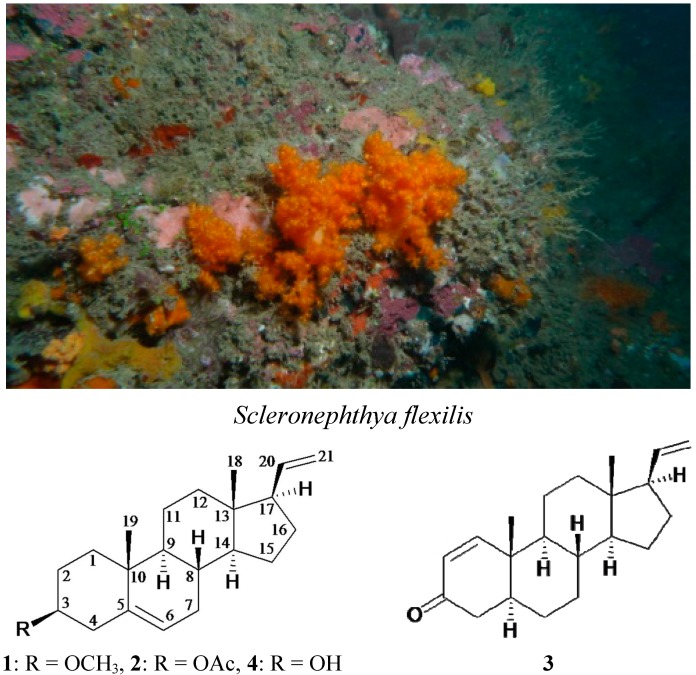
The soft coral *Scleronephthya flexilis* and the structures of 3β-methoxy-5,20-pregnadiene (**1**), 3β-acetoxy-5,20-pregnadiene (**2**), 5α-pregna-1,20-dien-3-one (**3**) and 3β-hydroxy-5,20-pregnadiene (**4**).

Octocorals belonging to the *Scleronephthya* genus have been well-recognized as marine organisms containing large quantities of steroids that exhibit varying degrees of bioactivities, such as cytotoxicity and anti-inflammatory activity [1,2,3,4,5,6,7]. In connection with our investigations of bioactive substances from marine organisms, a soft coral *Scleronephthya flexilis* (Thomson & Simpson, 1909, phylum Cnidaria, class Anthozoa, order Alcyonacea, family Nephtheidae) was selected for study (Scheme 1), as the ethyl acetate extract of this organism was found to exhibit cytotoxicity against the MOLT-4 human acute lymphoblastic leukemia (IC_50_ = 5.1 μg/mL). Our chemical investigation of this organism led to the isolation of a new metabolite, 3β-methoxy-5,20-pregnadiene (**1**), and two known analogues, 3β-acetoxy-5,20-pregnadiene (**2**) [2,6,8,9] and 5α-pregna-1,20-dien-3-one (**3**) [1,2,4,6,10,11,12,13,14,15,16,17] (Scheme 1). The structures of steroids **1**–**3** were elucidated on the basis of spectroscopic methods and by comparison of data with those of the related metabolites. The absolute configuration of steroid **2** was further confirmed by single-crystal X-ray diffraction analysis. The ability of steroid **3** to inhibit the growth of MOLT-4 cells was evaluated.

## 2. Results and Discussion

3β-Methoxy-5,20-pregnadiene (**1**) was found to have the molecular formula C_22_H_34_O (6° of unsaturation) by the HRESIMS at *m*/*z* 337.25027 (calcd for C_22_H_34_ONa, 337.25019). The ^13^C and DEPT spectra of **1** showed 22 carbon signals, including three methyls, eight sp^3^ methylenes, an sp^2^ methylene, five sp^3^ methines (including an oxymethine), two sp^2^ methines, two sp^3^ quaternary carbons and an sp^2^ quaternary carbon (Table 1). The ^1^H NMR spectra showed the presence of two tertiary methyls (δ_H_ 0.61, 1.01, each 3H, s), a methoxy group (δ_H_ 3.36, 3H, s), an oxymethine (δ_H_ 3.06, 1H, m), a vinyl group (δ_H_ 5.77, 1H, ddd, *J* = 16.8, 10.5, 7.7 Hz; 4.98, 1H, dd, *J* = 16.8, 1.4 Hz; 4.97, 1H, dd, *J* = 10.5, 1.4 Hz) and a trisubstituted olefin group (δ_H_ 5.36, 1H, br s) (Table 1). These spectroscopic data showed that **1** might have a pregnane skeleton.

**Table 1:** ^1^H (700 MHz, CDCl_3_) and ^13^C (175 MHz, CDCl_3_) NMR data for steroid **1**.

Position	δ_H_ (*J* in Hz)	δ_C_, Multiple
1	1.88 ddd (12.6, 4.2, 3.5), 1.05 m	37.3, CH_2_
2	1.92 ddd (12.6, 3.5, 2.8), 1.43 m	28.0, CH_2_
3	3.06 m	80.4, CH
4	2.39 (12.6, 4.2, 2.1), 2.16 m	38.7, CH_2_
5		141.0, C
6	5.36 br s	121.5, CH
7	2.01 m, 1.57 m	32.0, CH_2_
8	1.51 m	32.0, CH
9	0.96 m	50.5, CH
10		37.0, C
11	1.57 m, 1.48 dd (10.5, 5.6)	20.7, CH_2_
12	1.72 ddd (12.6, 4.2, 2.8), 1.05 m	37.4, CH_2_
13		43.4, C
14	1.04 m	56.0, CH
15	1.69 m, 1.21 dd (11.9, 5.6)	24.9, CH_2_
16	1.79 m, 1.57 m	27.2, CH_2_
17	1.96 m	55.4, CH
18	0.61 s	12.8, CH_3_
19	1.01 s	19.4, CH_3_
20	5.77 ddd (16.8, 10.5, 7.7)	139.8, CH
21	4.98 dd (16.8, 1.4), 4.97 dd (10.5, 1.4)	114.5, CH_2_
3-OCH_3_	3.36 s	55.6, CH_3_

**Figure 1:**
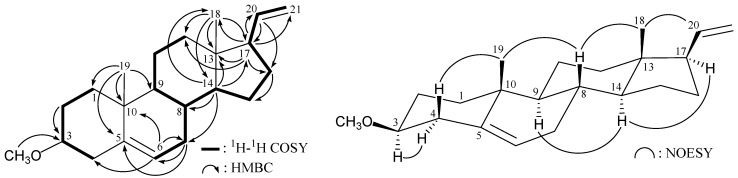
Selective key ^1^H–^1^H correlation spectroscopy (COSY), heteronuclear multiple-bond coherence (HMBC) and nuclear overhauser effect spectroscopy (NOESY) correlations for **1**.

The molecular skeleton of **1** was established by ^1^H−^1^H correlation spectroscopy (COSY) and heteronuclear multiple-bond coherence (HMBC) correlations as shown in Figure 1, in which C-3 (δ_C_ 80.4) was correlated with protons of a methoxy group (δ_H_ 3.36). Thus, similar to the structure of a known compound, 3β-hydroxy-5,20-pregnadiene (**4**) [8,9] (Scheme 1), **1** has a methoxy substituent at C-3. The relative stereochemistry of **1** was elucidated by correlations in a nuclear Overhauser effect spectroscopy (NOESY) experiment (Figure 1). The NOESY correlations between H_3_-18/H-20, H_3_-18/H-8, and H-8/H_3_-19 revealed the β-orientations of H-8, H_3_-18, H_3_-19, and the vinyl group at C-17, and α-orientations of H-9, H-14 and H-17. It was found that one of the methylene protons at C-4 (δ_H_ 2.39) exhibited a correlation with H_3_-19, and therefore it was assigned as H-4β, and the other C-4 proton (δ_H_ 2.16) as H-4α. There was a correlation between H-4α and H-3 (δ_H_ 3.06), indicating that the methoxy group at C-3 was β-oriented. Based on above spectral evidence, the structure of **1** was established as 3β-methoxy-5,20-pregnadiene.

**Figure 2:**
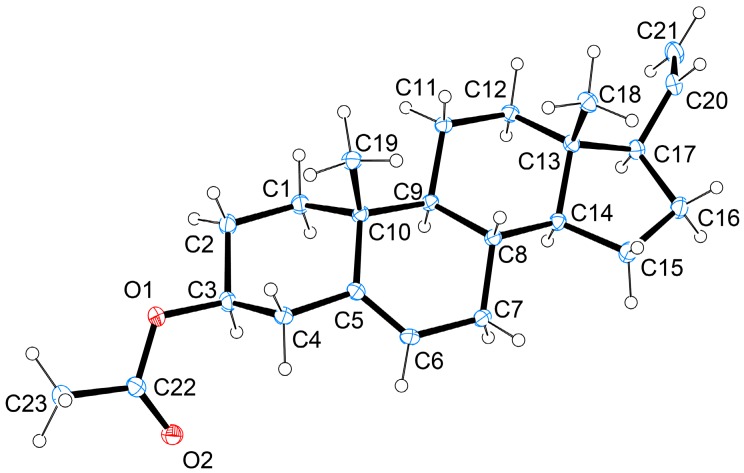
Molecular plot of **2** with confirmed absolute configuration.

Steroid **2** (3β-acetoxy-5,20-pregnadiene) was first obtained by chemical conversion from pregna-5,20-dien-3β-ol (**4**) in 1948 by Julian’s group [8] and the natural steroid **2** was isolated from the octocorals *Scleronephthya* sp. [2] and *Scleronephthya gracillimum* [6]. Its structure, including the absolute configuration, was determined by X-ray diffraction analysis for the first time in this study (Figure 2).

A well-known marine origin steroid, 5α-pregna-1,20-dien-3-one (**3**), was obtained in this study. Steroid **3** was first isolated from an unidentified soft coral from Canton Island, Pacific Ocean [10], and this compound has been obtained from various octocorals such as *Alcyonium gracillimum* [11], *Capnella erecta* [12], *Capnella thyrsoidea* [13], *Scleronephthya pallida* [1], *Scleronephthya gracillimum* [4,6], *Scleronephthya* sp. [2], *Sinularia papillosa* [14], *Sinularia* sp. [15] and *Spongodes* sp. [16]. The ^1^H and ^13^C data of **3** were identical to those of known pregnanes described previously [2,10,11,12,13,14,17] confirming that **3** was 5α-pregna-1,20-dien-3-one.

The cytotoxicities of steroids **2** and **3** towards human leukemia cells, including MOLT-4 (acute lymphoblastic leukemia), HL-60 (acute promyelocytic leukemia) and K-562 (chronic myelogenous leukemia) cells, were studied, and the results are shown in Table 2. MOLT-4 was the cell line most sensitive to the cytotoxic effects of 5α-pregna-1,20-dien-3-one (**3**). This encouraged us to expand our cytotoxicity study with the aim of revealing the mechanism of action of **3** against leukemia cancer cell lines, which we pursued in the current study.

**Table 2:** Cytotoxic data of steroids **2** and **3**.

Compounds	Cell Lines IC_50_ (g/mL)		
	MOLT-4	HL-60	K-562
**2**	NA	NA	NA
**3**	2.15	3.14	8.32
Doxorubicin ^a^	0.004	0.001	0.15

^a^ Doxorubicin was used as a positive control and NA = not active at 20 μg/mL for 72 h.

**Figure 3:**
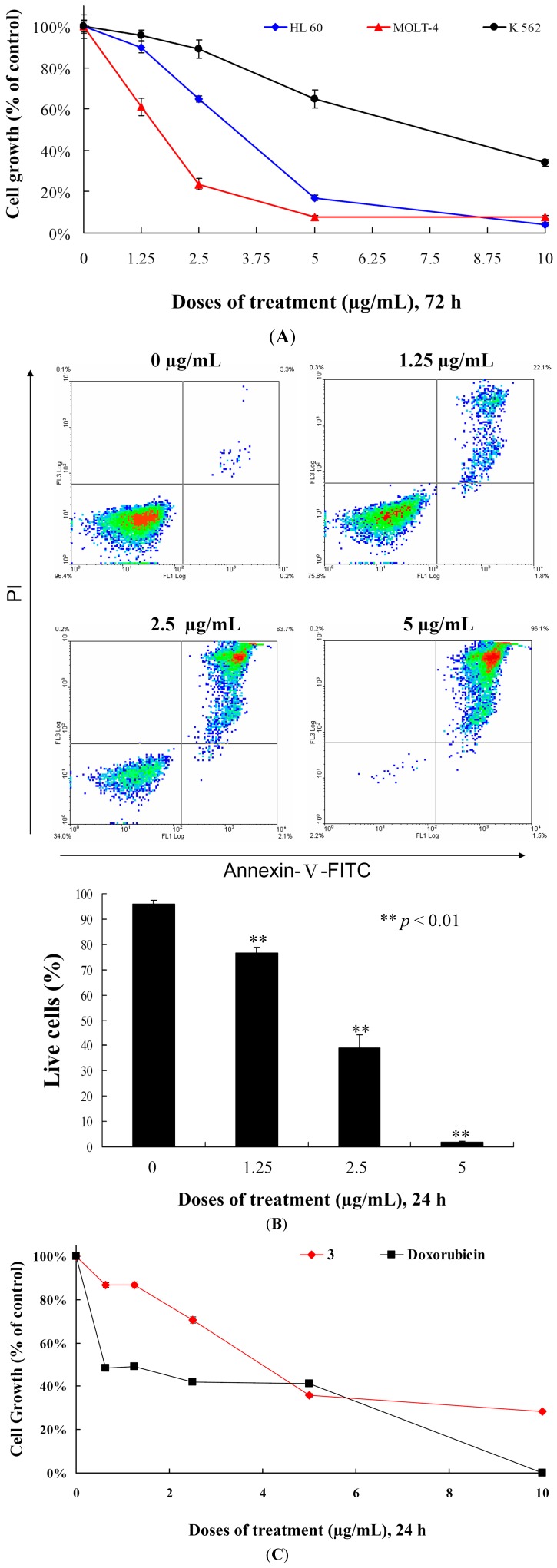
Cytotoxic and apoptotic effects of 5α-pregna-1,20-dien-3-one (**3**) on leukemia cells. (**A**) Leukemia cells were treated with varying concentrations of **3** for 72 h. Cell growth was assayed by MTT methods; (**B**) MOLT-4 cells were treated with varying concentrations of **3** for 24 h, then labeled with annexin V-FITC and PI (propidium iodide) and analyzed using flow cytometry; and (**C**) The viability of normal rat PBMCs were determined with different doses of **3** and doxorubicin. Results are presented as mean ± SD of three independent experiments.

We determined the effects of **3** treatment on the cell growth of different leukemia cell lines. Initially, we determined the IC_50_ values of **3** against MOLT-4, HL-60 and K-562 cells after 72 h and found that the cell growth of MOLT-4, HL-60 and K-562 cells were inhibited in dose-dependent manner with the IC_50_ values of 2.15, 3.14 and 8.32 μg/mL, respectively (Figure 3A and Table 2). HL-60 and K-562 cell lines are p53-negative cell lines [18]. MOLT-4 cells, originally derived from the same patient as MOLT-3 cells, are lymphoblastoid T cells and express normal p53 [19]. In addition, the cell growth of different leukemia cells was significantly suppressed by **3** treatment in a dose-dependent manner, regardless of p53 status (Figure 3A). We then evaluated whether the cytotoxicity of **3** is associated with apoptosis by examining the effect of **3** on cells stained with annexin V-FITC and propidium iodide (PI). As shown in Figure 3B, treatment with **3** at concentrations of 0, 1.25, 2.5 and 5 μg/mL for 24 h increased the percentages of annexin-positive cells from 4.1% to 23.3%, 61.1% and 98.2% as compared with the control group in MOLT-4 cells, respectively. To determine whether the cytotoxic effect of **3** is specific for cancer cells, we examined the effect of **3** on the viability of normal rat peripheral blood mononuclear cells (PBMC). At the highest dose (10 μg/mL), **3** treatment caused 71.6% suppression in the viability of PBMCs, nevertheless, doxorubicin treatment induced 99.9% suppression for 24 h (Figure 3C). Compared with PBMCs, **3** suppressed 99.9% of cell growth in MOLT-4 cells for 24 h. Thus, it is concluded that cytotoxic effect of **3** is more sensitive towards MOLT-4 cells compared to the normal rat PBMCs. In addition, our result suggested that growth inhibition of **3** is mediated with induction of apoptosis and operated independently in P53 pathway.

**Figure 4:**
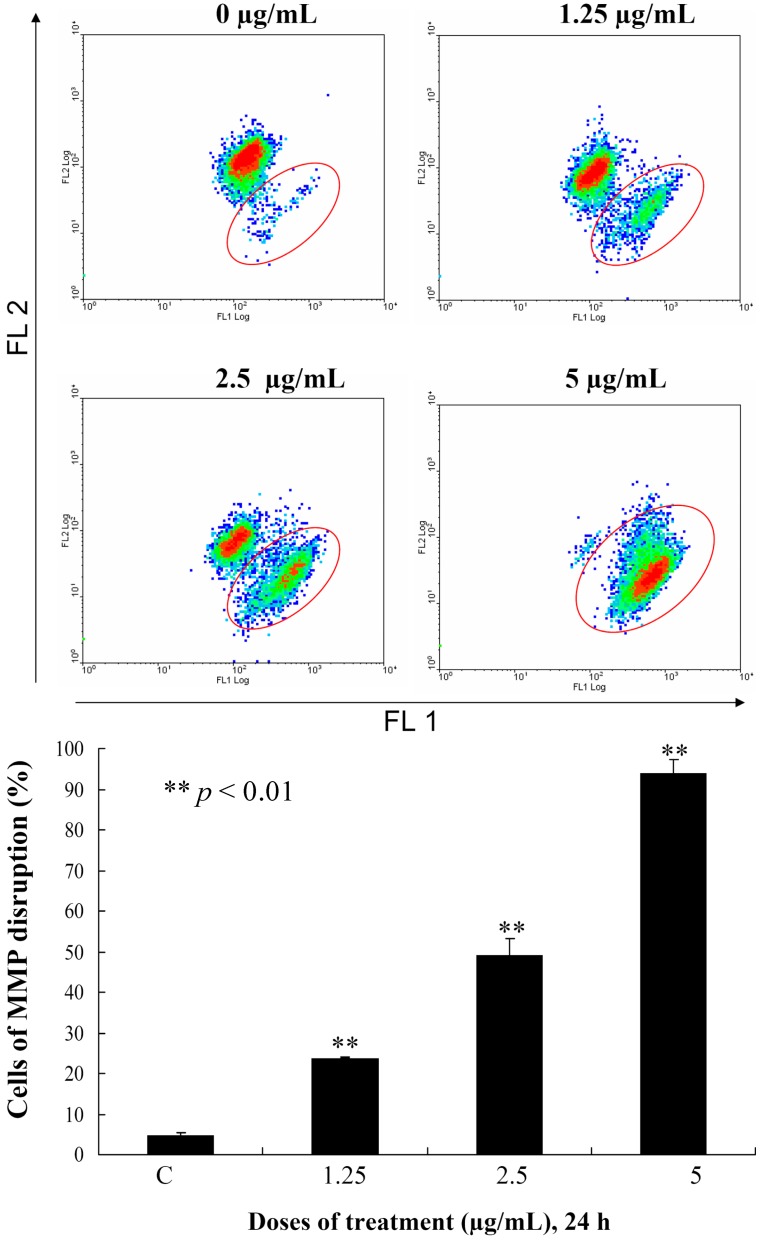
Flow cytometric results showing the effects of different concentrations of **3** (1.25, 2.5 and 5 μg/mL) on the disruption of the mitochondrial membrane potential (MMP).

This encouraged us to expand our cytotoxicity study with the aim of revealing the mechanism of action of **3**-induced leukemia MOLT-4 cells apoptosis. Mitochondria are organelles which play an important role in the life and death of the cells. Their importance is mainly attributed to energy production in the form of ATP. Additionally, mitochondrial dysfunction participates in the induction of apoptosis [20]. To examine whether the antiproliferative and apoptotic effects of **3** are involved mitochondrial dysfunction in MOLT-4 cells, flow cytometric assays with various fluorescent dyes were utilized. Different concentrations of **3** (0, 1.25, 2.5 and 5 μg/mL) were used for 24 h, and the change in the mitochondrial membrane potential (MMP) was analyzed. Treatment with **3** (1.25, 2.5 or 5 μg/mL) led to 23.8%, 49.2% and 93.8% disruption of the MMP, respectively, as detected using JC-1 cation dye in MOLT-4 cells (Figure 4). The recent result evidenced that one of **3** targets as an apoptosis inducer is to disrupt the bioenergetic steps of the mitochondria-medicated pathway.

## 3. Experimental Section

### 3.1. General Experimental Procedures

Optical rotations were measured using a Jasco P-1010 digital polarimeter (Japan Spectroscopic Corporation, Tokyo, Japan). Infrared spectra were recorded on a Varian Diglab FTS 1000 FT-IR spectrometer (Varian Inc., Palo Alto, CA, USA); peaks are reported in cm^−1^. NMR spectra were recorded on a Bruker AVIII HD 700X NMR spectrometer (Bruker, Bremen, Germany) or on a Varian Mercury Plus 400 NMR spectrometer (Varian Inc., Palo Alto, CA, USA) using the residual CHCl_3_ signal (δ_H_ 7.26 ppm) as the internal standard for ^1^H NMR and CDCl_3_ (δ_C_ 77.1 ppm) for ^13^C NMR. Coupling constants (*J*) are given in Hz. ESIMS and HRESIMS were recorded using a Bruker 7 Tesla solariX FTMS system (Bruker, Bremen, Germany). Column chromatography was performed on silica gel (230–400 mesh, Merck, Darmstadt, Germany). TLC was carried out on precoated Kieselgel 60 F_254_ (0.25 mm, Merck, Darmstadt, Germany); spots were visualized by spraying with 10% H_2_SO_4_ solution followed by heating. Normal-phase HPLC (NP-HPLC) was performed using a system comprised of a Hitachi L-7110 pump (Hitachi Ltd., Tokyo, Japan) and a Rheodyne 7725 injection port (Rheodyne LLC, Rohnert Park, CA, USA). Two normal-phase columns (Supelco Ascentis^®^ Si Cat #:581515-U, 250 mm × 21.2 mm, 5 µm; 581514-U, 250 mm × 10 mm, 5 µm, Sigma-Aldrich. Com., St. Louis, MO, USA) were used for NP-HPLC. Reverse-phase HPLC (RP-HPLC) was performed using a system comprised of a Hitachi L-7100 pump (Hitachi Ltd., Tokyo, Japan), a Hitachi L-2455 photodiode array detector (Hitachi Ltd., Tokyo, Japan), a Rheodyne 7725 injection port (Rheodyne LLC, Rohnert Park, CA, USA) and a reverse column (Varian Polaris C18-A, 250 mm × 10 mm, 5 µm; Varian Inc., Palo Alto, CA, USA).

### 3.2. Animal Material

Specimens of the octocoral *Scleronephthya flexilis* were collected by hand using scuba equipment off the coast of Southern Taiwan in September 2012, and stored in a freezer (−20 °C) until extraction. A voucher specimen (NMMBA-TWSC-12009) was deposited in the National Museum of Marine Biology and Aquarium, Pingtung, Taiwan.

### 3.3. Extraction and Isolation

Specimens of the soft coral *Scleronephthya flexilis* (wet weight 1.5 kg, dry weight 562 g) were minced and extracted with ethyl acetate (EtOAc). The EtOAc extract remaining after removal of the solvent (6.7 g) was separated by silica gel and eluted using *n*-hexane/EtOAc in a stepwise fashion from 100:1–pure EtOAc to yield 10 fractions A–J. Fraction C (514 mg) was chromatographed on silica gel using a mixture of *n*-hexane and EtOAc in a stepwise fashion from 45:1 to 10:1 to obtain 10 subfractions C1–C10. Fraction C3 (100 mg) was purified by NP-HPLC using a mixture of *n*-hexane and acetone (100:1) to obtain 8 subfractions C3A–C3H. Fraction C3F (3.1 mg) was repurified by NP-HPLC using a mixture of *n*-hexane and dichloromethane (5:2, flow rate: 1.0 mL/min) to yield 3β-methoxy-5,20-pregnadiene (**1**) (0.6 mg, *t*_R_ = 45 min). Fraction C4 (48 mg) was purified by NP-HPLC using a mixture of *n*-hexane and EtOAc (18:1) to obtain 10 subfractions C4A–C4J. Fraction C4F (38 mg) was repurified by RP-HPLC using a mixture of methanol and H_2_O (98:2, flow rate: 2.0 mL/min) to yield 3β-acetoxy-5,20-pregnadiene (**2**) (17.1 mg, *t*_R_ = 40 min). Fractions D and E were combined (2.76 g) and separated by Sephadex LH-20 using a mixture of dichloromethane and methanol (1:1) to obtain 13 subfractions D1–D13. Fractions D10 and D11 were combined and separated by NP-HPLC using a mixture of *n*-hexane and acetone (10:1) to obtain 8 subfractions D10A–D10H. Fraction D10B was further separated by NP-HPLC, using a mixture of *n*-hexane and dichloromethane (1:1, flow rate: 1.0 mL/min) to yield 5α-pregna-1,20-dien-3-one (**3**) (92.7 mg, *t*_R_ = 26 min).

3β-Methoxy-5,20-pregnadiene (**1**): 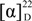 −6 (*c* 0.2, CHCl_3_); IR (neat) ν_max_ 1638 cm^−1^; ^1^H (700 MHz, CDCl_3_) and ^13^C (175 MHz, CDCl_3_) NMR data, see Table 1; ESIMS: *m*/*z* 337 [M + Na]^+^; HRESIMS: *m*/*z* 337.25027 (calcd for C_22_H_34_ONa, 337.25019).

3β-Acetoxy-5,20-pregnadiene (**2**): colorless prisms; mp 143−145 °C; 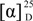 −60 (*c* 0.2, CHCl_3_); IR (neat) ν_max_ 1727 cm^−1^; ^1^H NMR (400 MHz, CDCl_3_) δ_H_ 5.76 (1H, ddd, *J* = 16.4, 11.2, 7.6 Hz, H-20), 5.38 (1H, br d, *J* = 5.6 Hz, H-6), 4.98 (1H, dd, *J* = 11.2, 0.8 Hz, H-21), 4.97 (1H, dd, *J* = 16.4, 0.8 Hz, H-21'), 4.60 (1H, m, H-3), 2.32 (2H, m, H_2_-4), 2.03 (3H, s, acetate methyl), 2.01 (1H, m , H-7), 1.98 (1H, m, H-17), 1.88 (1H, m, H-1), 1.86 (1H, m, H-2), 1.79 (1H, m, H-16), 1.73 (1H, m, H-12), 1.70 (1H, m, H-15), 1.60 (1H, m, H-2'), 1.58 (1H, m, H-7'), 1.57 (1H, m, H-16'), 1.54 (1H, m, H-11), 1.46 (1H, m, H-8), 1.42 (1H, m, H-11'), 1.19 (1H, m, H-15'), 1.16 (1H, m, H-1), 1.09 (1H, dd, *J* = 13.2, 4.0 Hz, H-12'), 1.03 (3H, s, H_3_-19), 1.01 (1H, m, H-14), 0.98 (1H, m, H-9), 0.61 (3H, s, H_3_-18); ^13^C NMR (100 MHz, CDCl_3_) δ_C_ 170.5 (C, acetate carbonyl), 139.8 (CH-20), 139.7 (C-5), 122.5 (CH-6), 114.5 (CH_2_-21), 73.9 (CH-3), 55.8 (CH-14), 55.3 (CH-17), 50.3 (CH-9), 43.4 (C-13), 38.1 (CH_2_-4), 37.3 (CH_2_-12), 37.0 (CH_2_-1), 36.7 (C-10), 32.0 (CH-8), 32.0 (CH_2_-7), 27.8 (CH_2_-2), 27.2 (CH_2_-16), 24.9 (CH_2_-15), 21.4 (CH_3_, acetate methyl), 20.6 (CH_2_-11), 19.3 (CH_3_-19), 12.7 (CH_3_-18); ESIMS: *m*/*z* 365 [M + Na]^+^; HRESIMS: *m*/*z* 365.2452 (calcd for C_23_H_34_O_2_Na, 365.2451).

5α-Pregna-1,20-dien-3-one (**3**): white powder; mp 116−118 °C; 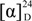 +45 (*c* 2.3, CHCl_3_); IR (neat) ν_max_ 1682 cm^−1^; ESIMS: *m*/*z* 321 [M + Na]^+^. The ^1^H and ^13^C NMR data of **3** were in full agreement with those of reported previously [2,10,11,12,13,14,17].

### 3.4. X-ray Crystallographic Analysis of Compound 2

Suitable colorless prisms of **2** were obtained from a solution of acetone. Crystal data and experimental details: C_23_H_34_O_2_, *M*_r_ = 342.50, crystal size 0.21 mm × 0.18 mm × 0.17 mm, crystal system orthorhombic, space group *P*2_1_2_1_2_1_ (#19), with *a* = 7.3639(4) Å, *b* = 11.5782(6) Å, *c* = 22.7667(11) Å, *V* = 1941.10(17) Å^3^, *Z* = 4, *D*_calcd_ = 1.172 g/cm^3^ and λ (Cu, k_α_) = 1.54178 Å. Intensity data were measured on a Bruker APEX-II CCD diffractometer equipped with a micro-focus Cu radiation source and Montel mirror up to θ_max_ of 66.7° at 100 K. All 12,991 reflections were collected. The structure was resolved by direct methods and refined by a full-matrix least-squares procedure. The refined structure model converged to a final *R*1 = 0.0279, *wR*2 = 0.0717 for 3358 observed reflections (*I* > 2σ(*I*)) and 229 variable parameters. The absolute configuration was determined by Flack’s method, with Flack’s parameter determined to be 0.09(6) [21,22].

### 3.5. Bioassay Material

RPMI-1640 medium, fetal calf serum (FCS), tryptan blue, penicillin G and streptomycin were obtained from Gibco BRL (Gaithersburg, MD, USA). 3-(4,5-Dimethylthiazol-2-yl)-2,5-diphenyltetrazolium bromide (MTT), dimethyl sulfoxide (DMSO) and all other chemicals were purchased from Sigma-Aldrich (St. Louis, MO, USA).

### 3.6. MTT Antiproliferative Assay

MOLT-4 (human acute lymphoblastic leukemia), HL-60 (human acute promyelocytic leukemia) and K-562 (human chronic myelogenous leukemia) cells were obtained from the American Type Culture Collection (ATCC, Manassas, VA, USA). Rat PBMCs were generated as described previously [23]. Cells were maintained in RPMI-1640 medium supplemented with 10% FCS, 2 mM glutamine, and antibiotics (100 units/mL penicillin and 100 μg/mL streptomycin) at 37 °C in a humidified atmosphere of 5% CO_2_. Cells were seeded at 4 × 10^4^ per well in 96-well culture plates before treatment with different concentrations of the tested compounds. The compounds were dissolved in DMSO (less than 0.02%) and made immediately to 1.25, 2.5, 5, 10 and 20 μg/μL prior to the experiments. After treatment for 72 h, the cytotoxicities of the tested compounds were determined using a MTT cell proliferation assay (thiazolyl blue tetrazolium bromide, Sigma-M2128). The MTT is reduced by the mitochondrial dehydrogenases of viable cells to a purple formazan product. The MTT-formazan product was dissolved in DMSO. Light absorbance values (OD = OD_570_ − OD_620_) were recorded at wavelengths of 570 and 620 nm using an ELISA reader (Anthos labtec Instrument, Salzburg, Austria) to calculate the concentration that caused 50% inhibition (IC_50_), *i.e.*, the cell concentration at which the light absorbance value of the experimental group was half that of the control group. These results were expressed as a percentage of the control ± SD established from *n* = 4 wells per one experiment from three separate experiments.

### 3.7. Annexin V/PI Apoptosis Assay

The externalization of phosphatidylserine (PS) and the membrane integrity were quantified using an annexin V-FITC staining kit (Strong Biotech Corporation, Taipei, Taiwan). In brief, 10^6^ cells were grown in 35-mm-diameter plates and were labeled with annexin V-FITC (10 μg/mL) and PI (20 μg/mL) prior to harvesting. After labeling, all plates were washed with a binding buffer and harvested. Cells were resuspended in the binding buffer at a concentration of 2 × 10^5^ cells/mL before analysis by a flow cytometer FACS-Calibur (Becton-Dickinson, San Jose, CA, USA) and CellQuest software. Approximately 10,000 cells were counted for each determination.

### 3.8. Determination of Mitochondrial Membrane Potential Disruption

These assays were performed as described previously. MMP disruption was detected with JC-1 cationic dye (5 μg/mL) [24]. In brief, the treated cells were labeled with a specific fluorescent dye for 30 min. After labeling, cells were washed with PBS and resuspended in PBS at a concentration of 1 × 10^6^ cells/mL before analysis by flow cytometry.

### 3.9. Statistics

The results were expressed as mean ± standard deviation (SD). Comparison in each experiment was performed using an unpaired Student’s *t*-test, and *p* values lower than 0.05 were considered to be statistically significant. (* *p* < 0.05; ** *p* < 0.01).

## 4. Conclusions

In the first study on the chemical constituents of soft coral *Scleronephthya flexilis*, three pregnane-type steroids, including a new metabolite, 3β-methoxy-5,20-pregnadiene (**1**), along with two known compounds, 3β-acetoxy-5,20-pregnadiene (**2**) and 5α-pregna-1,20-dien-3-one (**3**), were isolated. The structure of new steroid **1** was elucidated on the basis of spectroscopic methods. The absolute configuration of steroid **2** was further confirmed by X-ray diffraction analysis for the first time. Our results also suggest that the use of steroid **3** induced MMP disruption, as well as apoptosis in Molt-4 cells. The current work clearly supports the potential application of **3** for leukemia therapy. The soft coral *Scleronephthya flexilis* has been transplanted to culturing tanks located in the National Museum of Marine Biology and Aquarium, Taiwan, for extraction of additional natural products to establish a stable supply of bioactive material.

## Supplementary Files

Supplementary File 1 Supplementary Information (PDF, 1706 KB)
