# Supplementary material for: Pregnane-Type Steroids from the Formosan Soft Coral Scleronephthya flexilis
Source: Int J Mol Sci. 2014 Jun 6;15(6):10136–49. doi: 10.3390/ijms150610136 (PMC4100144; doi:10.3390/ijms150610136)
Supplement: Supplementary File 1 — Supplementary Information (PDF, 1706 KB) [file ijms-15-10136-s001.pdf]

Supplementary Information

Figure S1. ^{1}H NMR spectrum (700 MHz) of compound **1** in CDCl3.

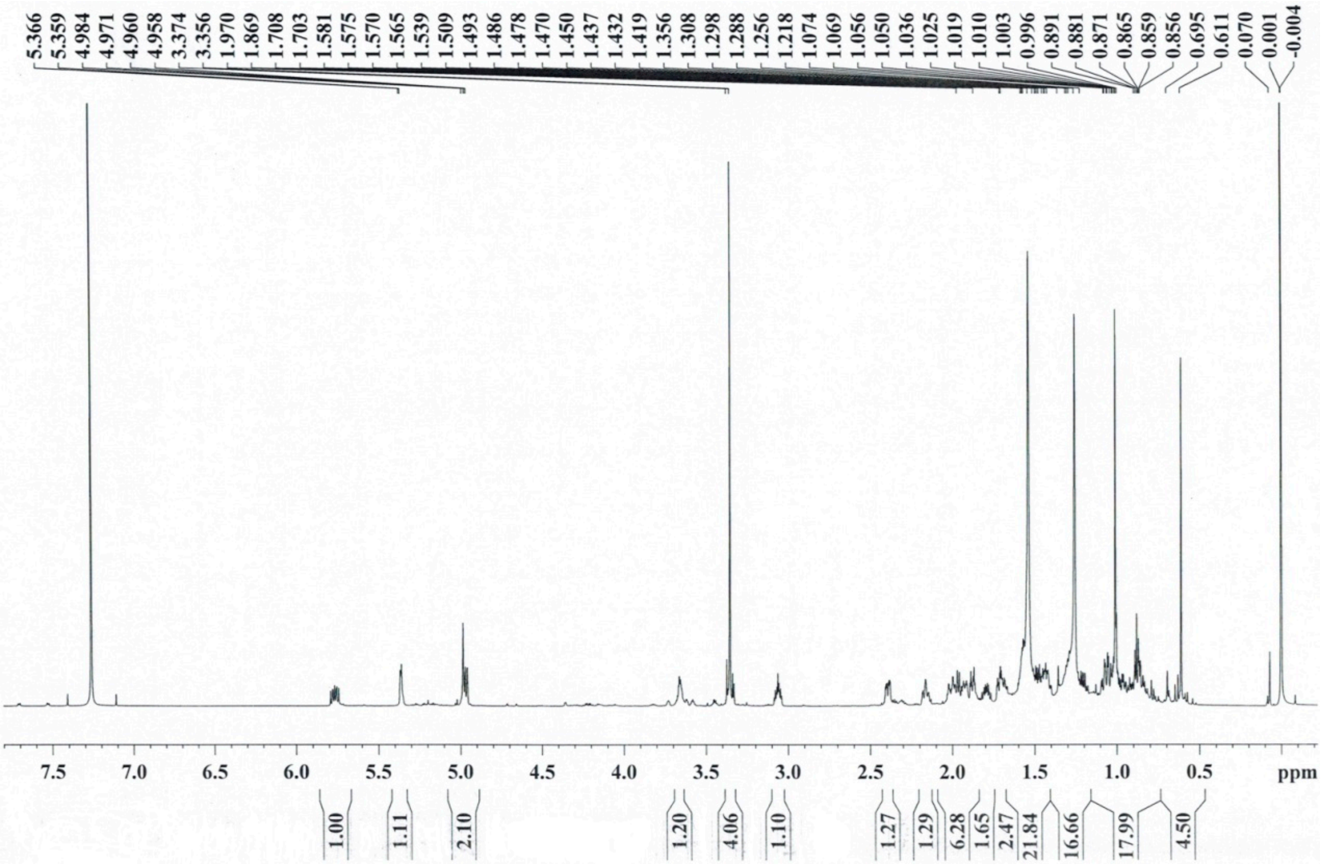

Figure S2. ^{13}C NMR spectrum (175 MHz) of compound **1** in $CDCl_3$.

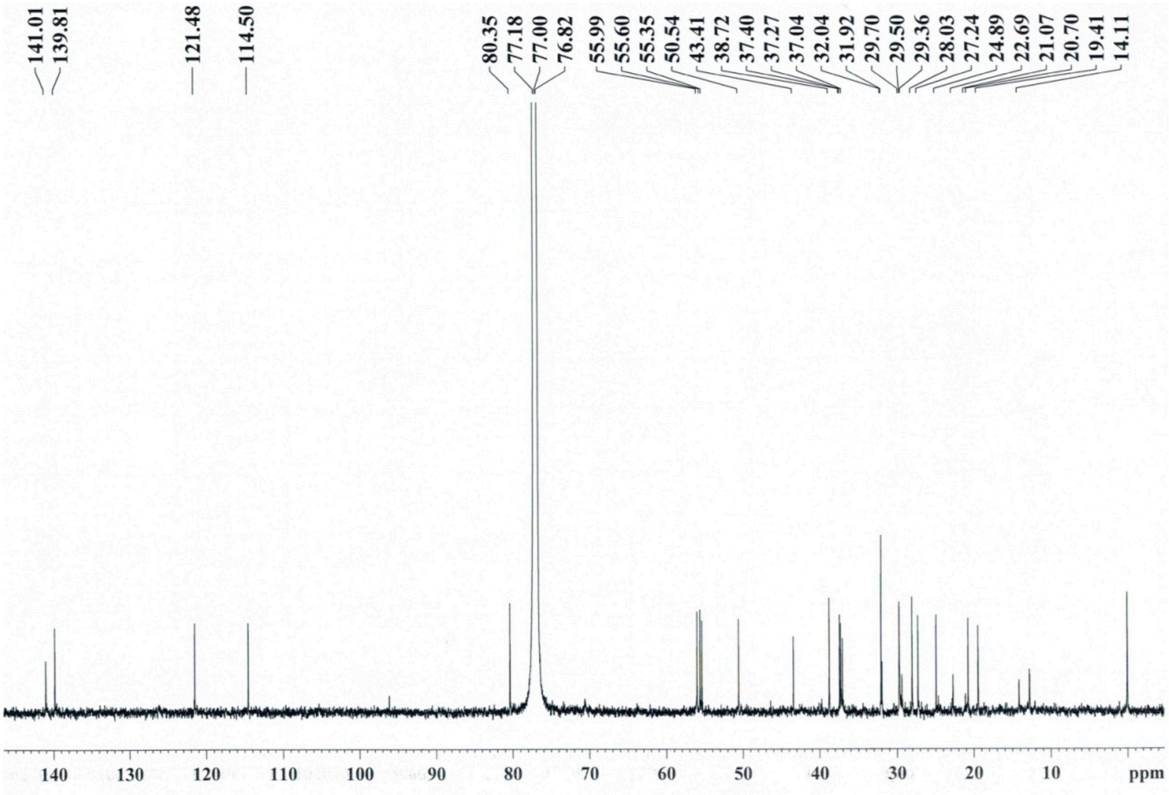

Figure S3. DEPT spectrum (700 MHz) of compound **1** in $CDCl_3$.

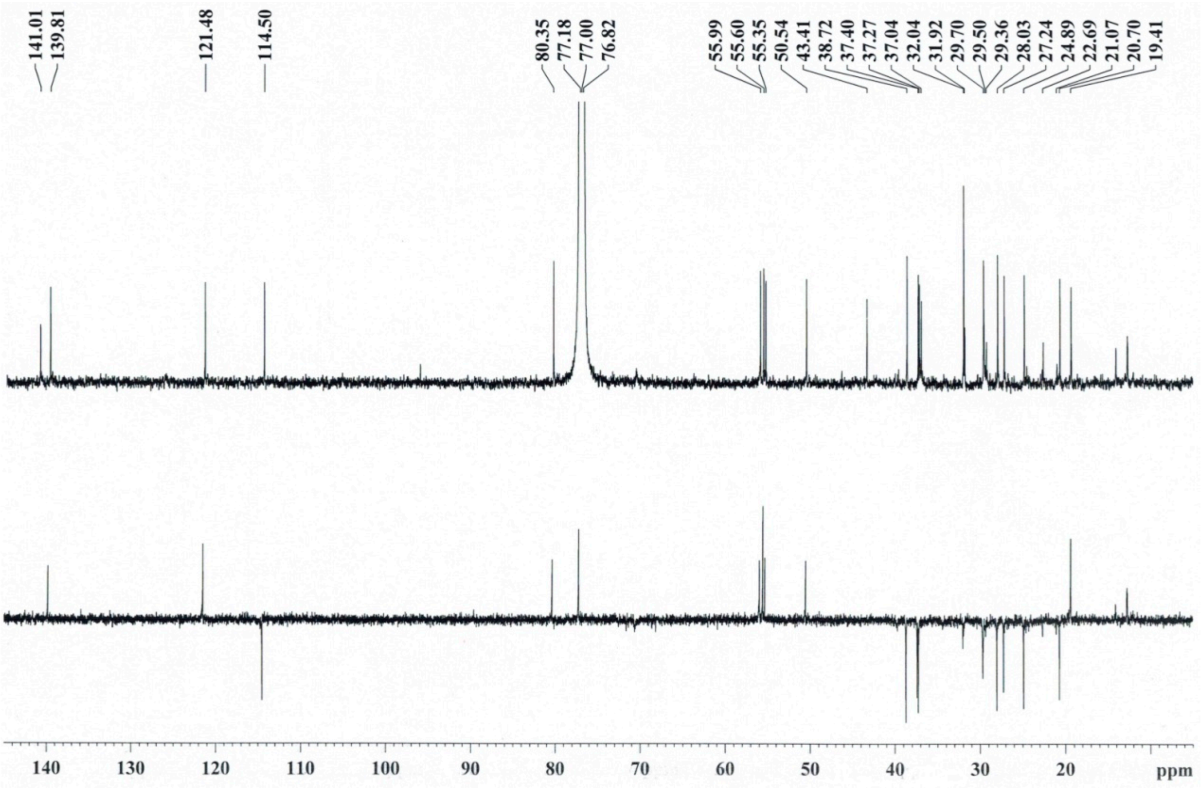

Figure S4. HSQC spectrum (700 MHz) of compound **1** in $CDCl_3$.

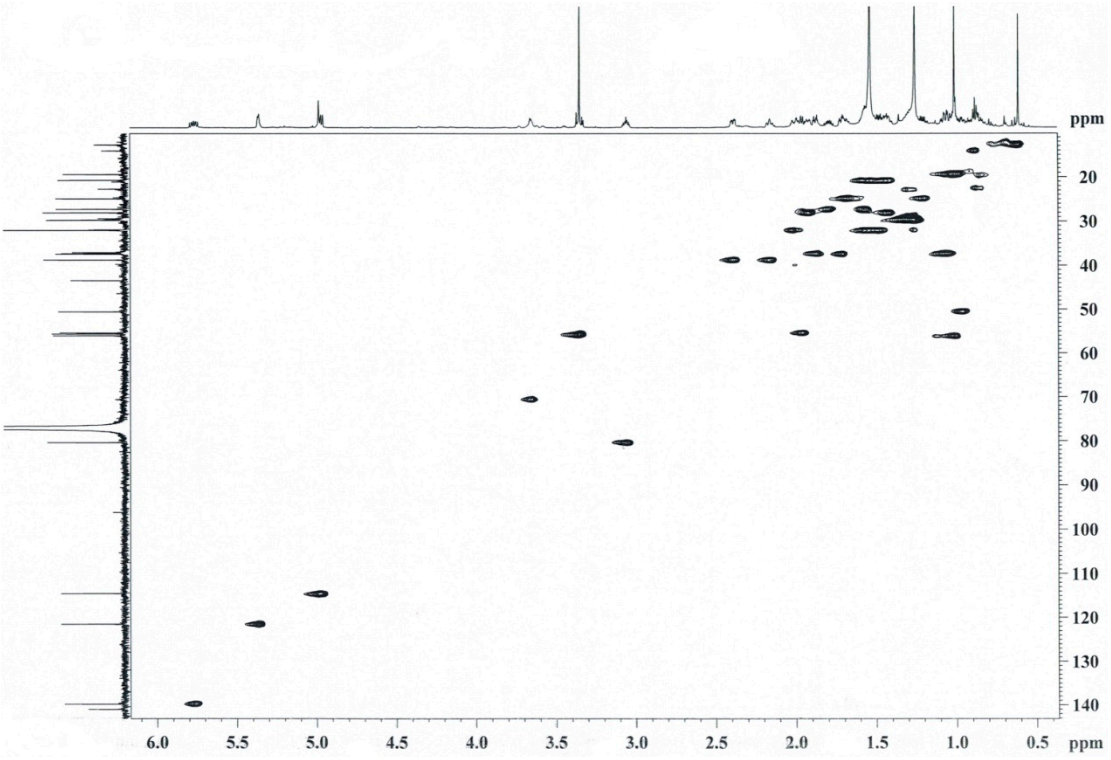

Figure S5. HMBC spectrum (700 MHz) of compound **1** in $CDCl_3$.

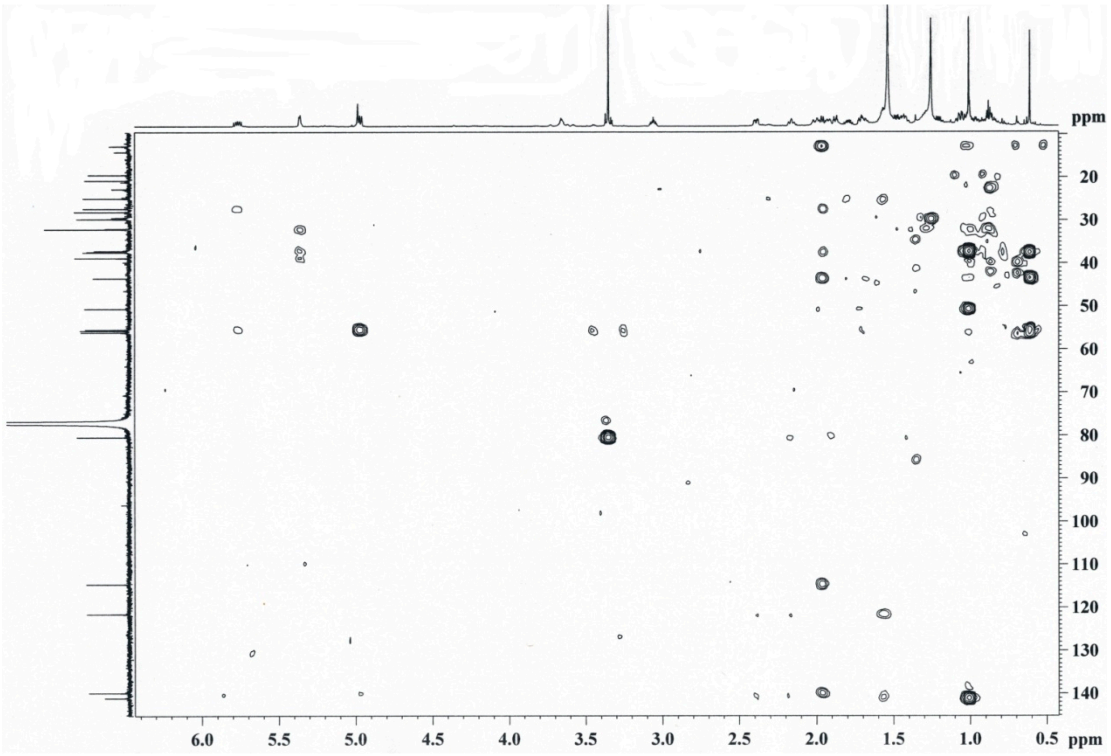

Figure S6. COSY spectrum (700 MHz) of compound **1** in $CDCl_3$.

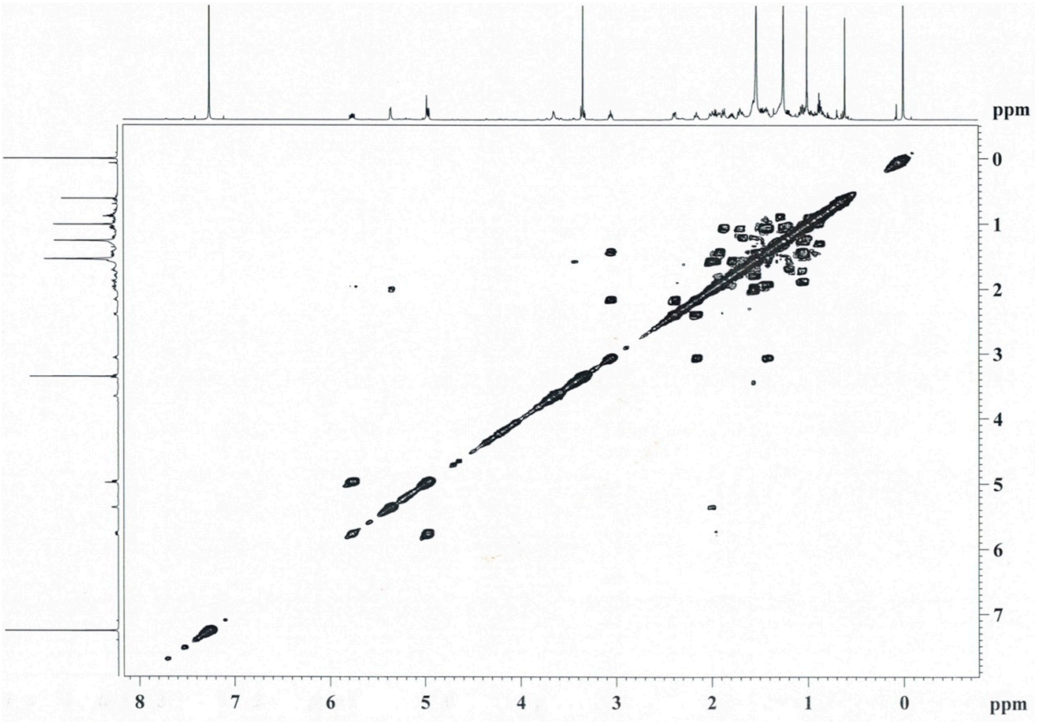

Figure S7. NOESY spectrum (700 MHz) of compound **1** in $CDCl_3$.

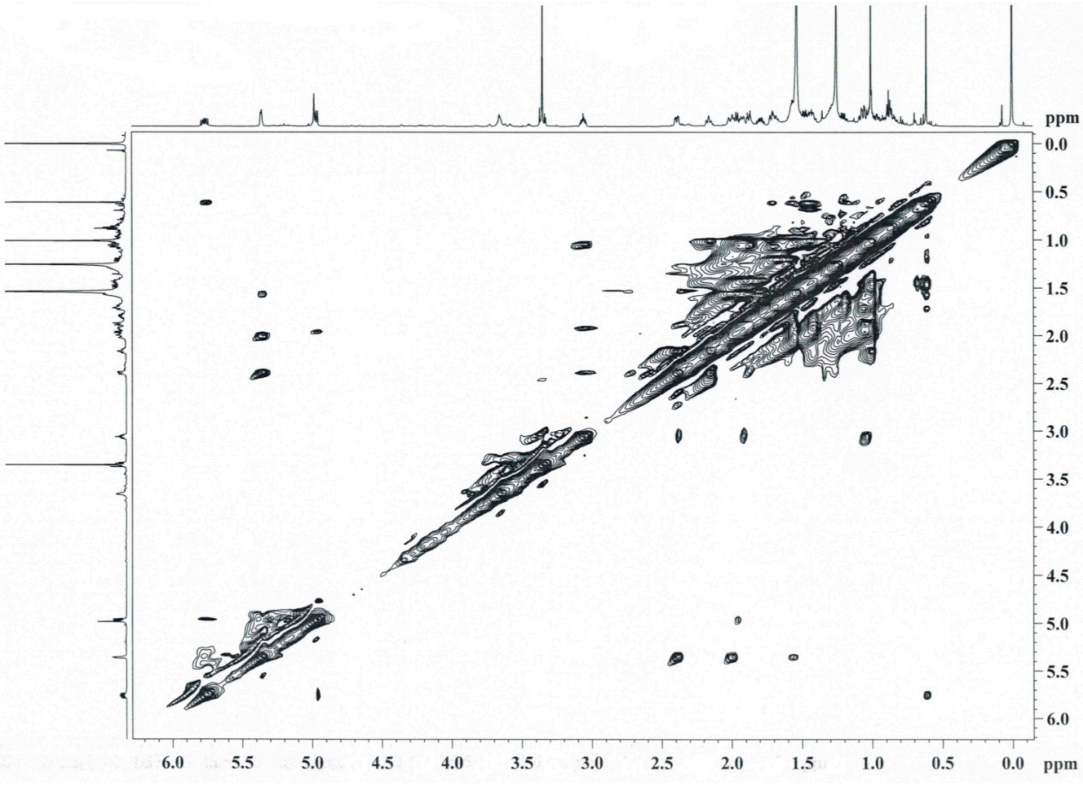

© 2014 by the authors; licensee MDPI, Basel, Switzerland. This article is an open access article distributed under the terms and conditions of the Creative Commons Attribution license (http://creativecommons.org/licenses/by/3.0/).
